# CD25 downregulation by tumor exosomal microRNA‐15a promotes interleukin‐17‐producing γδ‐T‐cells‐mediated radioresistance in nasopharyngeal carcinoma

**DOI:** 10.1002/mco2.70078

**Published:** 2025-02-02

**Authors:** Xiwei Wang, Zheng Xiang, Yanmei Zhang, Chloe Ran Tu, Chunyu Huang, Yuet Chung, Wenyue Zhang, Manni Wang, Yinping Liu, Wenwei Tu

**Affiliations:** ^1^ Department of Paediatrics and Adolescent Medicine Li Ka Shing Faculty of Medicine University of Hong Kong Hong Kong SAR China; ^2^ CAS Key Laboratory of Quantitative Engineering Biology Shenzhen Institute of Synthetic Biology Shenzhen Institute of Advanced Technology Chinese Academy of Sciences Shenzhen China; ^3^ Department of Microbiology and Immunology Health Science Center (School of Medicine) Jinan University Jinan China; ^4^ Department of data sciences Dana‐Farber Cancer Institute Harvard University Boston Massachusetts USA; ^5^ Shenzhen Key Laboratory for Reproductive Immunology of Peri‐implantation Shenzhen Zhongshan Institute for Reproduction and Genetics Shenzhen Zhongshan Obstetrics & Gynecology Hospital (formerly Shenzhen Zhongshan Urology Hospital) Shenzhen China

**Keywords:** exosomes, interleukin‐17‐producing γδ‐T cells, microRNA‐15a, nasopharyngeal carcinoma, radioresistance

## Abstract

Interleukin (IL)‐17‐producing γδ‐T cells (γδT‐17) are a major source of IL‐17 within the tumor microenvironment and have been shown to influence tumor development and therapy outcomes in various cancers. However, the role and presence of γδT‐17 cells in nasopharyngeal carcinoma (NPC) remain poorly understood. It is also unclear how these cells might affect radiotherapy, the primary treatment for NPC patients. In this study, we discovered that NPC tumor tissues were rich in γδT‐17 cells. Exosomes released from NPC cells (NPC‐Exos) could direct γδ‐T cells to differentiate into γδT‐17 cells. These NPC‐Exos‐induced γδT‐17 cells were found to enhance radioresistance in NPC, both in vitro and in vivo. Blocking IL‐17 secreted by NPC‐Exos‐induced γδT‐17 cells restored NPC cell sensitivity to radiation and elevated radiation‐induced cell death. Mechanistic studies revealed that NPC‐Exos not only increased the release of IL‐17‐promoting cytokines IL‐1β, IL‐6, and IL‐23 from dendritic cells, but also suppressed CD25/IL‐2 signaling in γδ‐T cells, facilitating γδT‐17 differentiation. The suppression of CD25/IL‐2 signaling was driven by microRNA‐15a (miR‐15a) carried by NPC exosomes. Furthermore, miR‐15a inhibitors were able to prevent γδT‐17 induction by NPC‐Exos. Our findings reveal a novel immunoregulatory role of NPC‐Exos and offer potential strategies to combat NPC radioresistance.

## Graphical abstract

Graphical abstract

1. NPC cells secrete exosomes containing abundant miR‐148a.

2. NPC exosomes induced γδT‐17 cells by promoting IL‐17‐driving cytokines and downregulating CD25.

3. γδT‐17 cells promote radioresistance.

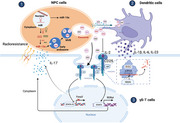

## 1 INTRODUCTION

Nasopharyngeal carcinoma (NPC) is a highly prevalent form of Epstein–Barr virus (EBV)‐associated cancer, especially in Southeast Asia.^1^ Radiotherapy is the primary treatment for early‐stage NPC, achieving a 5‐year survival rate of about 85%. However, most NPC patients are asymptomatic during the early stages and are often diagnosed at more advanced stages, where the likelihood of therapeutic failure due to radioresistance is higher.^2^ Radioresistance is a significant clinical challenge, leading to local recurrence and distant metastasis, thus diminishing the effectiveness of radiotherapy. This resistance arises from the tumor's ability to reduce sensitivity to radiation or adapt to radiotherapy‐induced damage, typically through mechanisms such as enhanced DNA repair or upregulation of anti‐apoptotic proteins.^3^, ^4^

NPC cells employ several intrinsic mechanisms to evade radiotherapy‐induced cell death. Key among these are enhanced DNA repair mechanisms, such as homologous recombination and non‐homologous end‐joining, which allow the tumor cells to repair radiation‐induced DNA double‐strand breaks more efficiently than normal cells.^5^ Additionally, the upregulation of anti‐apoptotic proteins like Bcl‐2 and surviving can prevent apoptosis, enabling the cells to survive despite radiation.^6^ Another significant contributor to radioresistance is the epithelial‐to‐mesenchymal transition, where epithelial NPC cells acquire mesenchymal traits, which not only enhance their metastatic potential but also make them more resistant to radiation‐induced cell death.^7^ Understanding these mechanisms is essential for developing new strategies to counteract radioresistance.

Beyond tumor‐intrinsic factors, the tumor microenvironment (TME) plays a crucial role in fostering radioresistance. Chronic inflammation within the TME is strongly associated with tumor development and resistance to radiotherapy.^8^, ^9^ Immune cells, including macrophages, dendritic cells (DCs), and various T‐cell subsets, contribute to this inflammatory environment, releasing cytokines that promote tumor survival and resistance.^10^ Among these cytokines, interleukin (IL)‐17 has gained attention for its role in promoting tumor growth and immune evasion.^11^ IL‐17 is primarily secreted by T cells, including αβ‐T and γδ‐T cells. γδ‐T cells are innate‐like T cells that play critical roles in cancer biology and therapy.^12^, ^13^, ^14^, ^15^ IL‐17‐producing γδ‐T cells (γδT‐17 cells), in particular, are known to contribute significantly to the inflammatory milieu of the TME.^16^, ^17^ Despite this, the exact mechanisms through which γδT‐17 cells contribute to NPC radioresistance remain poorly understood and warrant further investigation.

Current efforts to overcome radioresistance in NPC involve several therapeutic strategies. One promising approach is targeting the DNA repair machinery with agents such as poly (ADP‐ribose) polymerase inhibitors, which prevent tumor cells from repairing radiation‐induced DNA damage, thereby enhancing the cytotoxic effects of radiotherapy.^18^ Additionally, the combination of radiotherapy with chemotherapy agents such as cisplatin has been shown to improve outcomes by increasing DNA damage and preventing tumor recurrence, though toxicity remains a significant concern.^18^ Another emerging strategy is the use of immune checkpoint inhibitors, such as anti‐PD‐1/PD‐L1 antibodies, to enhance the immune system's response to radiotherapy by inhibiting immune evasion mechanisms.^19^ These therapies aim to reduce radioresistance by either sensitizing the tumor cells or by boosting the immune system's ability to target resistant tumor cells.

Exosomes, small vesicles secreted by tumor cells, have recently been recognized as critical mediators in the TME, influencing tumor progression and immune evasion.^20^, ^21^ NPC‐derived exosomes (NPC‐Exos) are known to regulate immune responses by delivering microRNA (miRNA) and proteins that modulate T‐cell function and promote tumor survival.^22^, ^23^ For instance, our previous research demonstrated that NPC‐Exos induce IL‐6 production from macrophages, fostering a pro‐tumorigenic environment.^24^ NPC‐Exos have also been shown to inhibit Th1 differentiation and antitumor immunity through miRNA‐mediated mechanisms.^23^ In this study, we explore how NPC‐Exos can induce the differentiation of γδT‐17 cells, contributing to NPC radioresistance by enhancing the secretion of IL‐17 and upregulating anti‐apoptotic proteins. Understanding the role of exosomal miRNAs in regulating γδT‐17 cells could provide novel insights into overcoming radioresistance and improving therapeutic outcomes for NPC patients.

## 2 RESULTS

### 2.1 NPC‐Exos can induce γδT‐17 cells

**FIGURE 1:**
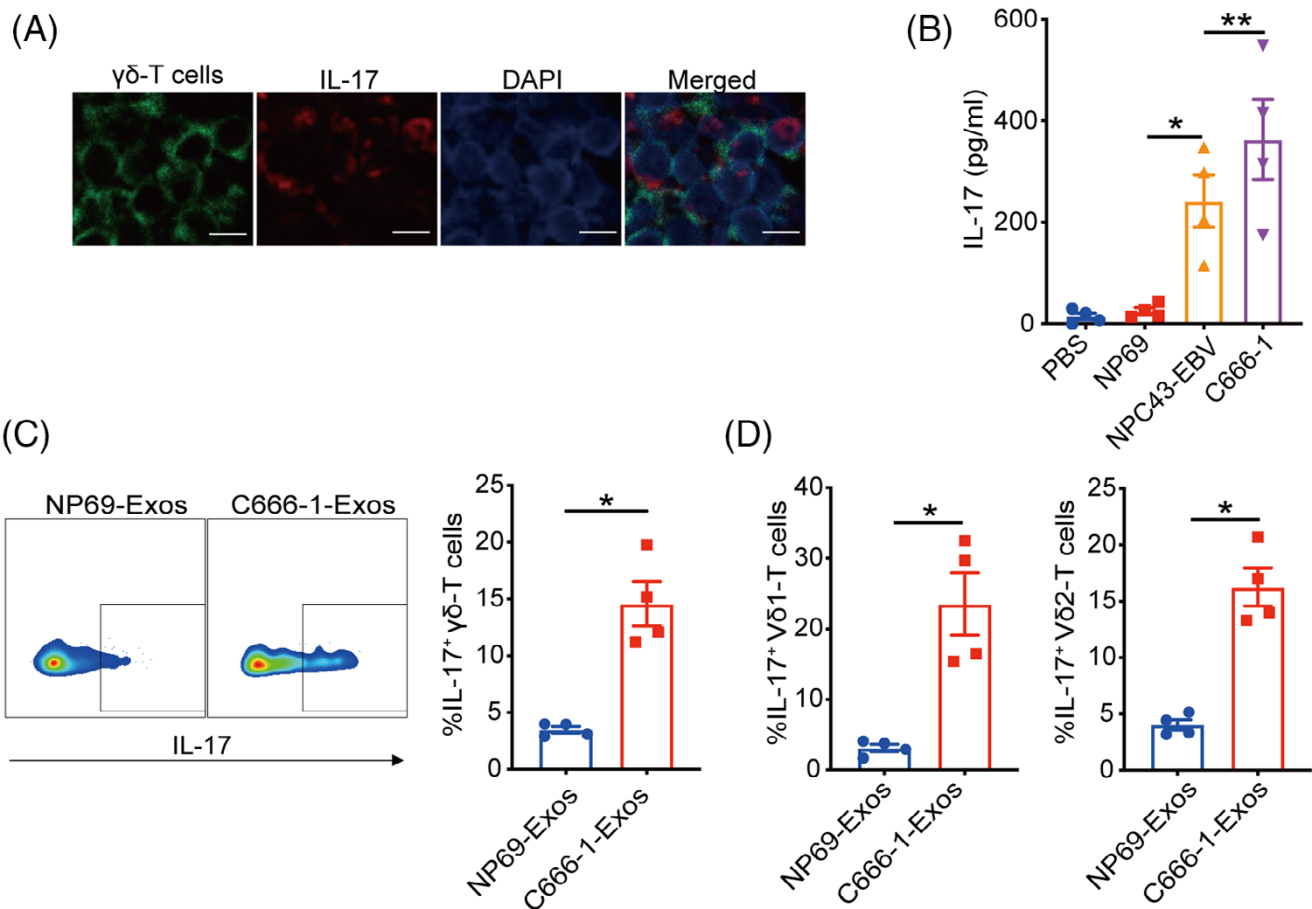
Exosomes released from nasopharyngeal carcinoma (NPC‐Exos) induce γδT‐17 cells in the tumor microenvironment. (A) γδ‐T cells in NPC tissues revealed by immunofluorescence analysis. Scale bar, 10 µm. (B) γδ‐T cells were co‐cultured with dendritic cells (DCs) in the presence of PBS, NP69‐Exos, or NPC‐Exos. Seven days later, the secretion of IL‐17 was detected. (C) Percentages of γδT‐17 cells induced by NP69‐Exos or C666‐1‐Exos. (D) Percentages of IL‐17^+^Vδ1‐ and IL‐17^+^Vδ2‐T cells induced by NP69‐Exos or C666‐1‐Exos. Quantitative data are shown as mean ± SEM of four biological replicates. *p < 0.05, **p < 0.01.

To investigate the existence of γδT‐17 cells, tumor tissues from NPC patients were collected and subjected to dual immunofluorescent analysis of IL‐17 and TCR‐γ/δ. In Figure 1A, cells co‐expressed IL‐17 and TCR‐γ/δ were found, suggesting the existence of γδT‐17 cells in NPC tumor tissues. Considering tumor cells‐derived exosomes play essential roles in regulating T‐cell differentiation and functions,^25^ we next determined the effects of NPC‐Exos on the induction of γδT‐17 cells. NPC‐Exos were isolated through differential ultracentrifugation and characterized as we described before.^24^ Compared with PBS (phosphate buffered saline) or exosomes derived from immortalized normal epithelial cells (NP69‐Exos), NPC‐Exos induced significantly more secretion of IL‐17 from γδ‐T cells than NP69‐Exos (Figure 1B). Intracellular staining analysis also confirmed the promotion of γδT‐17 cells by NPC‐Exos (Figure 1C and Figure S1). In addition, IL‐17 was upregulated by NPC‐Exos both in Vδ1 and Vδ2 subsets of γδ‐T cells (Figure 1D), which are the major subset of γδ‐T cells in human. These data demonstrate that NPC‐Exos can induce γδT‐17 cells.

### 2.2 NPC‐Exos‐induced‐γδT‐17 cells promote NPC radioresistance

**FIGURE 2:**
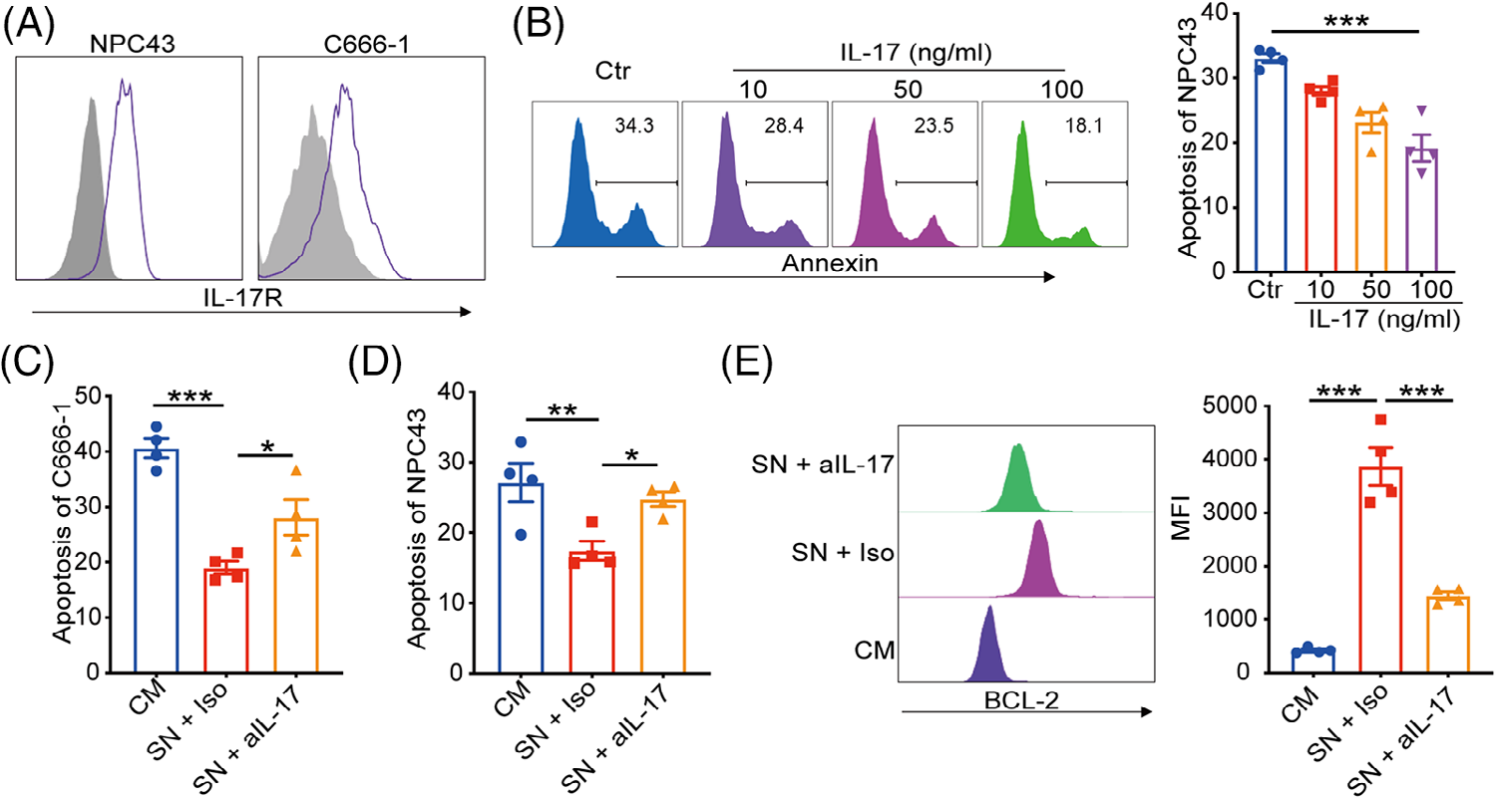
Exosomes released from nasopharyngeal carcinoma (NPC‐Exos)‐induced γδT‐17 cells promote NPC radioresistance in vitro. (A) Expression of IL‐17R on NPC cells. (B) NPC43 cells were irradiated at 3 Gy and cultured with recombinant IL‐17. PBS was used a control (Ctr). After 48 h, cell apoptosis was determined. (C and D) NPC cells were irradiated at 3 Gy and cultured with complete medium or the supernatant of NPC‐Exos‐induced γδT‐17 cells in the presence of neutralizing anti‐IL‐17 antibody or isotype control. After 48 h, C666‐1 (C) or NPC43 (D) cell apoptosis was determined. (E) C666‐1 cells were irradiated at 3 Gy and cultured with complete medium or the supernatant of C666‐1‐Exos‐induced γδT‐17 cells in the presence of neutralizing anti‐IL‐17 antibody or isotype control (Iso). After 24 h, BCL‐2 expression was determined. Quantitative data are shown as mean ± SEM of four biological replicates. *p < 0.05, **p < 0.01, ***p < 0.001. CM, complete medium; SN, supernatant.

**FIGURE 3:**
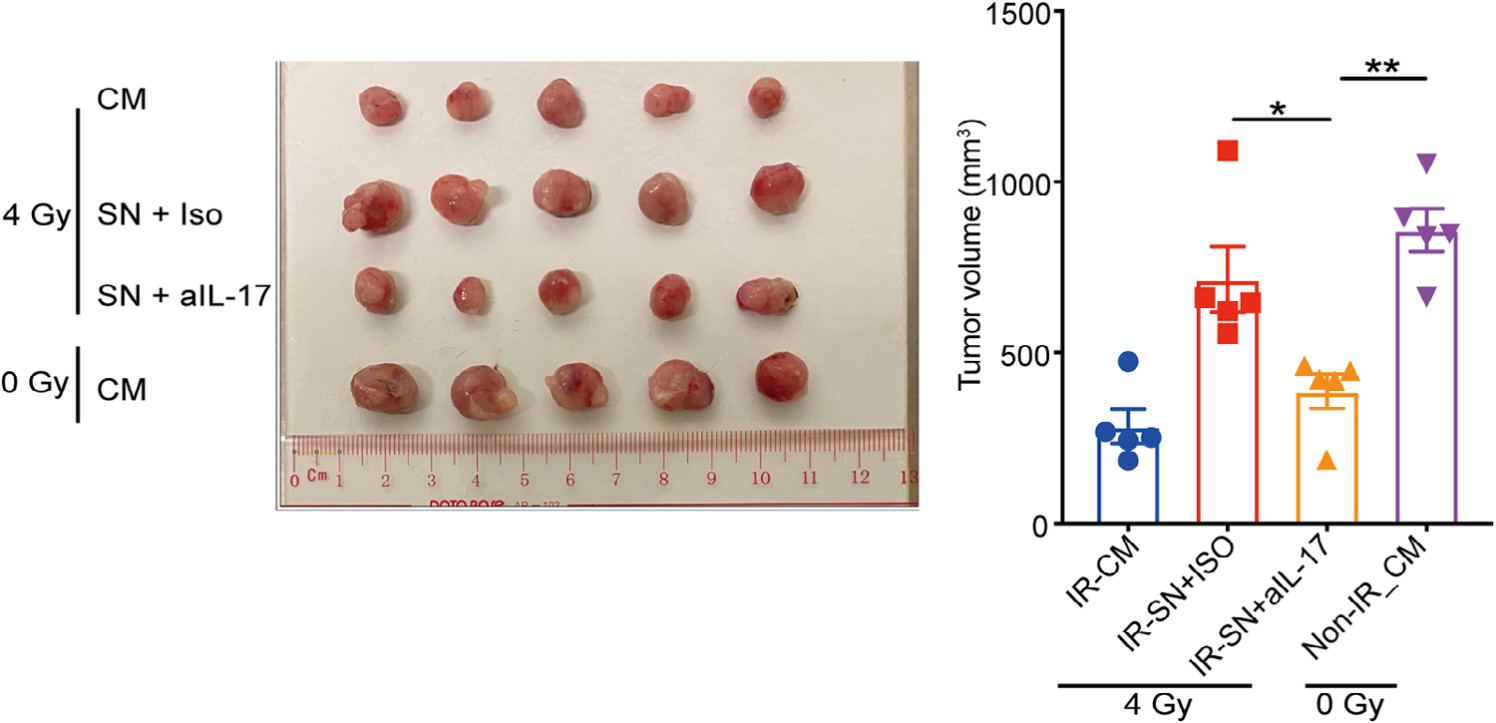
Exosomes released from nasopharyngeal carcinoma (NPC‐Exos)‐induced γδT‐17 cells promote NPC radioresistance in vivo. C666‐1 tumor‐bearing Rag2^−/−^γc^−/−^ mice were irradiated (IR) at 0 or 4 Gy and injected intraperitoneally with complete medium or the supernatant of NPC‐Exos‐induced γδT‐17 cells (*n* = 5). Three weeks later, tumors were excised and photographed. The data shown are representative of two independent experiments. *p < 0.05, **p < 0.01. CM, complete medium; SN, supernatant.

Considering IL‐17 is a critical cytokine initiating chronic inflammation that can affect the therapeutic responses of cancer cells, we further determined the effects of NPC‐Exos‐induced γδT‐17 cells on NPC radiotherapy. First, robust expression of the IL‐17 receptor was found on NPC tumor cells (Figure 2A), while expression of IL‐17 receptor on normal epithelial cells is less evident (Figure S2), providing the molecular basis to respond to NPC‐Exos‐induced γδT‐17 cells. Next, recombinant IL‐17 inhibited the radiation‐induced apoptosis of NPC cells in a dose‐dependent manner (Figure 2B). More importantly, the supernatant from NPC‐Exos‐induced γδT‐17 cells significantly reduced the radiation‐induced apoptosis of NPC cells. In contrast, blockage of IL‐17 in the supernatant restored radiosensitivity (Figure 2C,D). Further experiments found that IL‐17 secreted from γδT‐17 cells increased the expression of an anti‐apoptotic protein BCL‐2 (Figure 2E), which can promote radioresistance of cancer cells.^26^, ^27^ Furthermore, an in vivo study demonstrated that treatment with supernatant from NPC‐Exos‐induced γδT‐17 cells increased the radioresistance of NPC tumors. However, blockage of IL‐17 in the supernatant recovered the radiosensitivity of NPC tumors (Figure 3). Taken together, these data indicate that NPC‐Exos‐induced γδT‐17 cells can secrete IL‐17 to promote NPC radioresistance both in vitro and in vivo.

### 2.3 NPC‐Exos induce γδT‐17 cells by inducing IL‐17‐promoting cytokines from DCs

**FIGURE 4:**
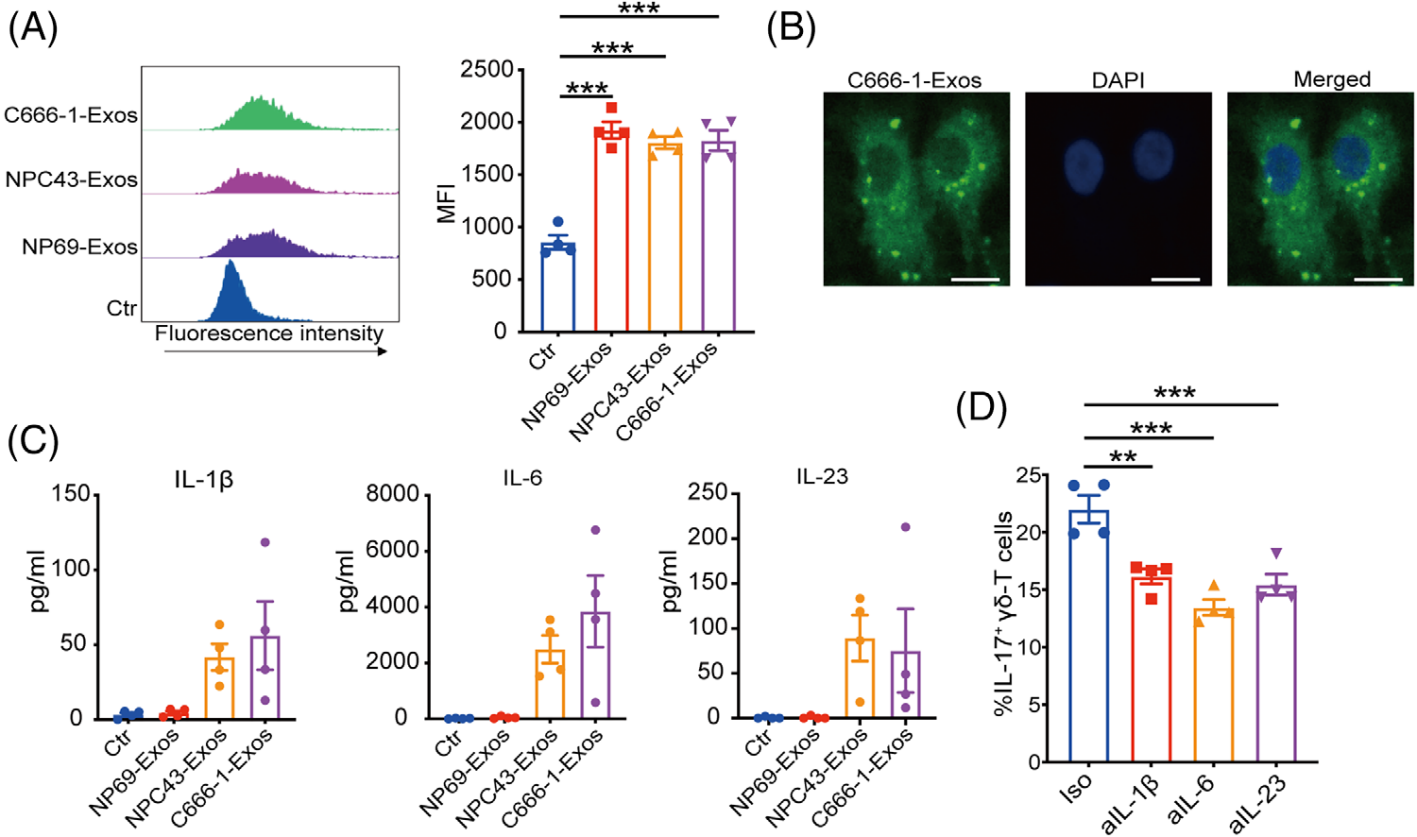
Exosomes released from nasopharyngeal carcinoma (NPC‐Exos) promote IL‐17‐driving cytokines from dendritic cells (DCs) to induce γδT‐17 cells. (A) Exosomes were labeled with carboxyfluorescein succinimidyl ester (CFSE) and used to treat DCs for 24 h. Pellets isolated from a non‐conditioned, exosome‐free medium were labeled with CFSE and served as control (Ctr). The median fluorescence intensity (MFI) of CFSE in DCs was determined by flow cytometry. (B) DCs were treated with CFSE‐labeled C666‐1‐Exos for 24 h; then, the fluorescent signal was analyzed by confocal microscopy. Scale bars, 5 µm. (C) DCs were treated with PBS or exosomes for 24 h, then the secretion of IL‐1β, IL‐6, or IL‐23 was detected. (D) γδ‐T cells and DCs were incubated with C666‐1‐Exos in the presence of neutralizing anti‐IL‐1β, IL‐6, IL‐23 antibodies or isotype (Iso) control antibody. Seven days later, expression of IL‐17 in γδ‐T cells was detected. Quantitative data are shown as mean ± SEM of four biological replicates. **p < 0.01, ***p < 0.001.

Next, we explored the mechanisms underlying the induction of γδT‐17 cells by NPC‐Exos. Since antigen‐presenting cells play critical roles in T‐cell differentiation, we determined the effects of NPC‐Exos on DCs. Data showed that NP69‐Exos were taken by DCs in similar efficacy with NPC‐Exos (Figure 4A), in which the cellular uptake was confirmed by confocal analysis (Figure 4B).

IL‐17‐promoting cytokines, such as IL‐1β, IL‐6, or IL‐23, can be secreted by DCs and promote the induction of IL‐17‐producing T cells.^28^, ^29^, ^30^ We found that NPC‐Exos induced significantly more IL‐17‐promoting cytokines from DCs than the exosomes derived from NP69 cells (NP69‐Exos; Figure 4C). Blockage of the IL‐17‐promoting cytokines IL‐1β, IL‐6, or IL‐23 suppressed the induction of γδT‐17 cells by NPC‐Exos (Figure 4D). These data demonstrate that NPC‐Exos can induce IL‐17‐promoting cytokines from antigen‐presenting cells to promote γδT‐17 cell induction.

### 2.4 NPC‐Exos promote γδT‐17 cells by blocking CD25/IL‐2 signaling

**FIGURE 5:**
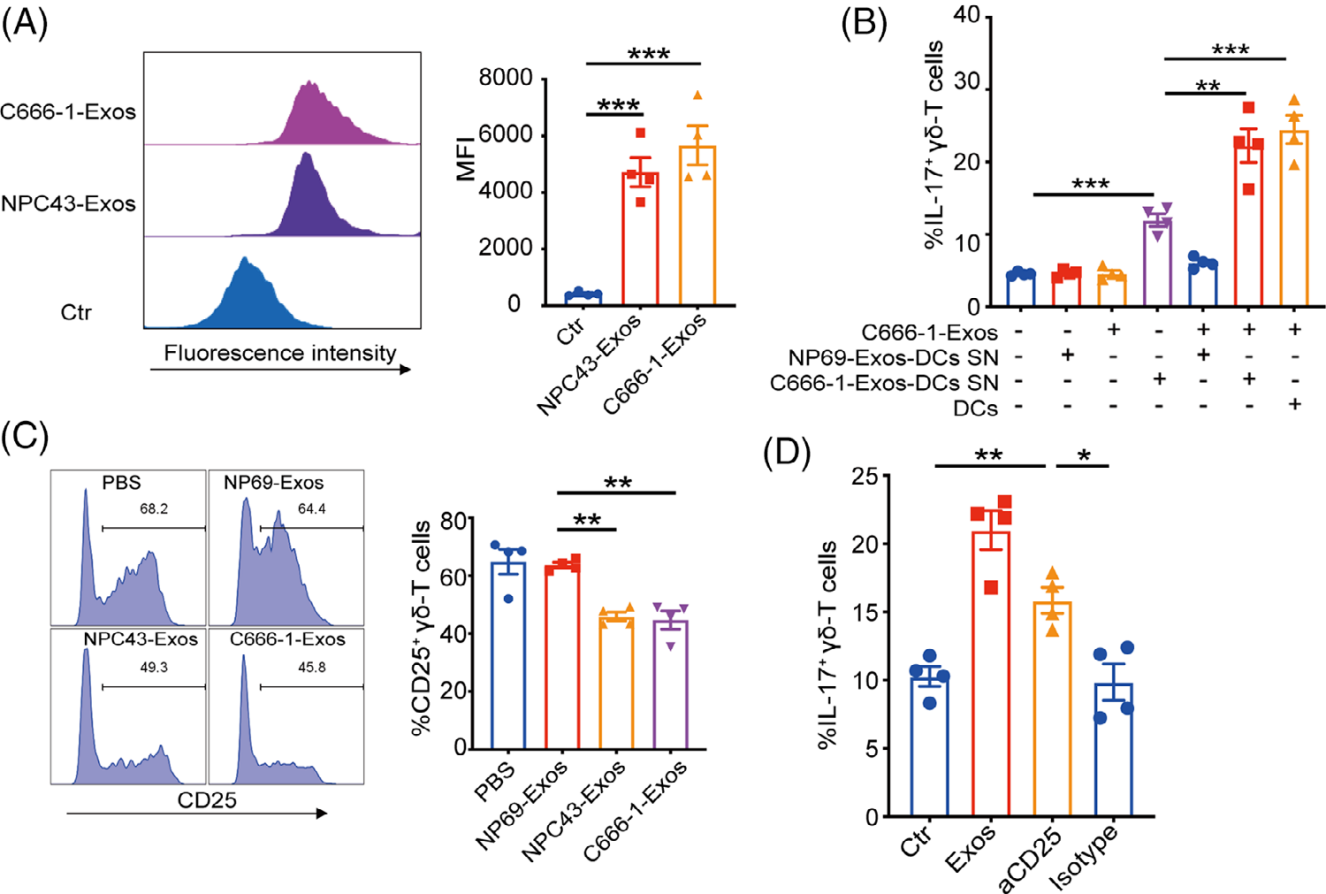
Exosomes released from nasopharyngeal carcinoma (NPC‐Exos) promote γδT‐17 cells by blocking the CD25 pathway. (A) NPC‐Exos were labeled with carboxyfluorescein succinimidyl ester (CFSE) and incubated with γδ‐T cells. Pellets isolated from a nonconditioned, exosome‐free medium were labeled with CFSE and served as control (Ctr). After 24 h, the fluorescence intensity of CFSE in γδ‐T cells was determined. (B) Purified γδ‐T cells were incubated with the supernatant from dendritic cells (DCs) pretreated with NP69‐Exos or C666‐1‐Exos in the presence of NP69‐Exos or C666‐1‐Exos. As a positive control, γδ‐T cells were co‐cultured with DCs and incubated with C666‐1‐Exos. Seven days later, the percentages of γδT‐17 cells were determined. (C) Purified γδ‐T cells were treated with NP69‐Exos or NPC‐Exos and cultured in the presence of anti‐CD3/CD28 beads. Forty‐eight hours later, the expression of CD25 on γδ‐T cells was detected. (D) γδ‐T cells were incubated with the supernatant from C666‐1‐Exos‐pretreated DCs, in the presence of C666‐1‐Exos, neutralizing anti‐CD25 or isotype control antibody. Culture with the supernatant from NP69‐Exos‐pretreated DC was used as control (Ctr). Seven days later, the percentages of γδT‐17 cells were determined. Quantitative data are shown as mean ± SEM of four biological replicates. *p < 0.05, **p < 0.01, ***p < 0.001. SN, supernatant.

Since tumor‐derived exosomes can also directly modulate T cells functions and differentiation,^22^, ^23^, ^31^ we then determined the direct effect of NPC‐Exos on γδT‐17 cells. Flow cytometry analysis revealed efficient uptake of NPC‐Exos by γδ‐T cells (Figure 5A). Furthermore, the supernatant from DCs pretreated with NPC‐Exos directly induced γδT‐17 cells. The coculture of γδ‐T cells with C666‐1‐Exos rather than NP69‐Exos further promoted the induction of γδT‐17 cells (Figure 5B). These data indicate that NPC‐Exos, but not NP69‐Exos, can polarize γδ‐T cells directly to synergize with IL‐17‐promoting cytokines in the induction of γδT‐17 cells.

As cytokine signaling plays critical roles in T‐cell differentiation, we further determined the effects of NPC‐Exos on the cytokine receptors related to the differentiation of IL‐17‐producing cells. The results showed that NPC‐Exos did not affect receptor expressions of IL‐1β, IL‐6, and IL‐23 (Figure S3) on γδ‐T cells. However, NPC‐Exos significantly decreased CD25 expression on γδ‐T cells (Figure 5C), which is an essential component of high‐affinity IL‐2 receptors. Although it remains unknown whether CD25/IL‐2 signaling affects γδT‐17 cells differentiation, previous studies have demonstrated that deficiency in CD25/IL‐2 signaling was associated with the differentiation of IL‐17‐producing T cells.^32^, ^33^ Therefore, our findings strongly implied that NPC‐Exos could directly inhibit CD25/IL‐2 signaling and synergize to induce γδT‐17 cells. We added excessive exogenous IL‐2 to γδ‐T cells to test this possibility by culturing them with the supernatant from NPC‐Exos‐pretreated DCs that contains IL‐17‐promoting cytokines. Treatment with IL‐2 resulted in a dose‐dependent inhibition of γδT‐17 cell induction (Figure S4). On the contrary, inhibition of the IL‐2 pathway by blocking CD25 had similar efficacy as NPC‐Exos in the induction of γδT‐17 cells (Figure 5D). Therefore, these results demonstrate that NPC‐Exos can promote γδT‐17 cells through ameliorating the suppressive effect of CD25/IL‐2 signaling on γδ‐T cells.

### 2.5 NPC exosomal miR‐15a promote γδT‐17 cells by downregulating CD25 expression

**FIGURE 6:**
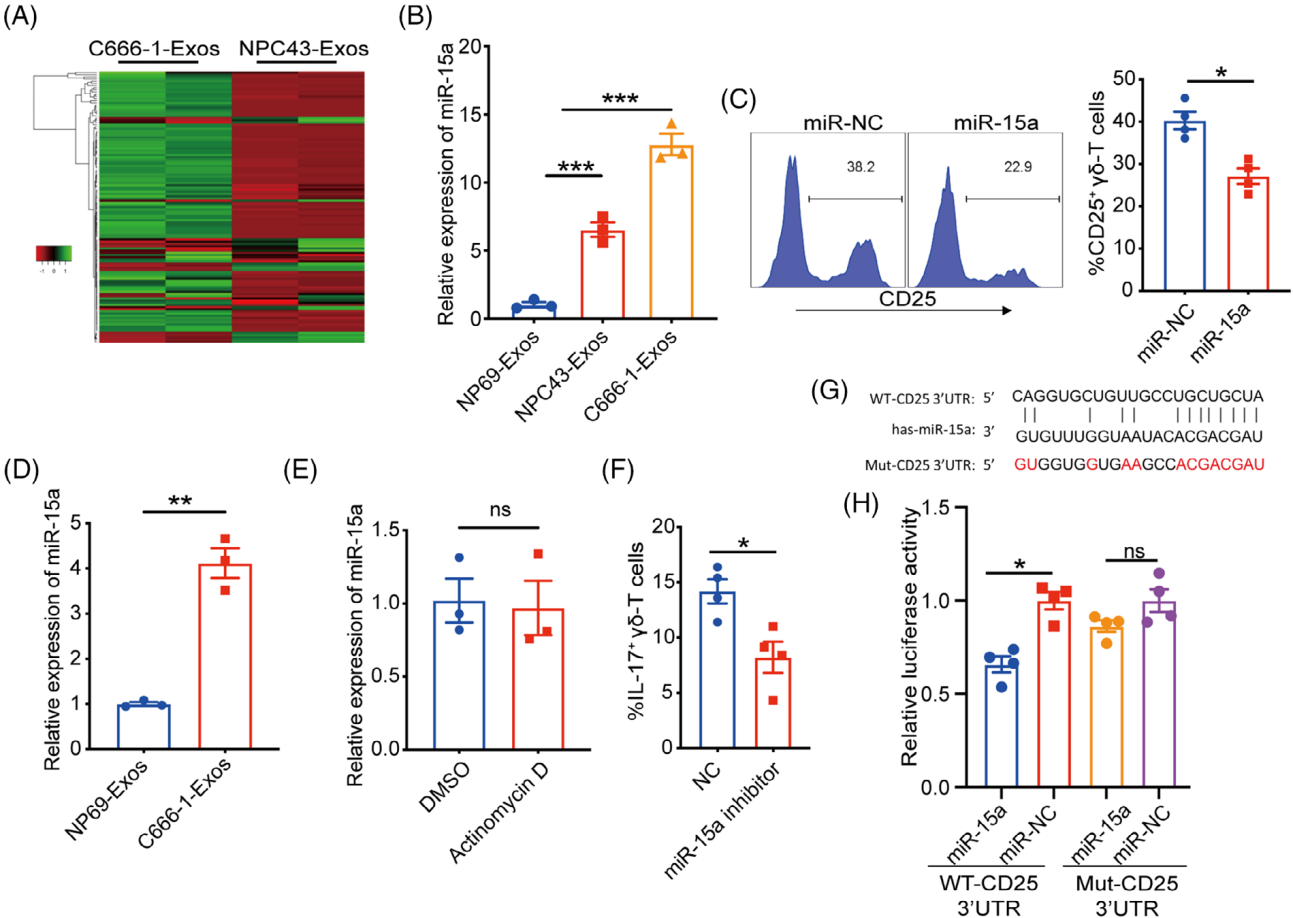
NPC exosomal miR‐15a promote γδT‐17 cells by downregulating CD25 expression. (A) miRNA profiles of NP69‐Exos and C666‐1‐Exos. (B) Relative expression of miR‐15a in NP69‐Exos and exosomes released from nasopharyngeal carcinoma (NPC‐Exos). (C) Purified γδ‐T cells were transfected with miR‐15a or negative control (NC) and cultured in the presence of anti‐CD3/CD28 beads. Forty‐eight hours later, the expression of CD25 on γδ‐T cells was detected. (D) Expression of miR‐15a in γδ‐T cells after being treated with NP69‐Exos or C666‐1‐Exos for 24 h. (E) γδ‐T cells were treated with C666‐1‐Exos and cultured with DMSO or actinomycin D. Twenty‐four hours later, expression of miR‐15a was determined. (F) γδ‐T cells were transfected with miR‐15a inhibitor or negative control (NC), then cultured in the supernatant from C666‐1‐Exos‐pretreated dendritic cells (DCs) and C666‐1‐Exos. Seven days later, the percentages of γδT‐17 cells were determined. (G) The wild‐type (WT) and mutant (Mut) binding sites of miR‐15a and the 3′UTR of the CD25 gene. (H) The binding of miR‐15a to 3′UTR of CD25 gene was investigated in HEK293T cells using a dual luciferase assay. Quantitative data are shown as mean ± SEM of three to four independent experiments or biological replicates. ns, not significant; *p < 0.05, **p < 0.01, ***p < 0.001.

Exosomes are the most critical circulating carriers of miRNA that are widely involved in immunoregulation. Therefore, we further explored whether the miRNA in NPC‐Exos mediated the downregulation of CD25 to promote γδT‐17 cells. Next‐generation sequencing analysis revealed distinct miRNA profiles between NP69‐Exos and C666‐1‐Exos (Figure 6A). Utilizing bioinformatic analysis (e.g., miRwalk database), we identified candidate miR‐15a that was upregulated in C666‐1‐Exos could regulate CD25 expression. RT‐PCR confirmed that miR‐15a was upregulated in NPC‐Exos compared with NP69‐Eoxs, consistent with the RNA sequencing analysis (Figure 6B).

Furthermore, we found that transfection of miR‐15a significantly inhibited the expression of CD25 on γδ‐T cells (Figure 6C). Then, we determined whether miR‐15a could be transferred from NPC‐Exos to γδ‐T cells for inhibiting CD25 expression. Data showed that the level of miR‐15a was upregulated in γδ‐T cells after being treated with NPC‐Exos (Figure 6D), and the inhibition of RNA synthesis did not change the miR‐15a profile in NPC‐Exos‐treated γδ‐T cells (Figure 6E), suggesting that the upregulated miR‐15a was attributed to the transfer of NPC exosomal miR‐15a rather than endogenous synthesis. Moreover, the transfection of miR‐15a inhibitor decreased the induction of γδT‐17 cells by NPC‐Exos (Figure 6F), further confirming that NPC exosomal miR‐15a mediated the induction of γδT‐17 cells. To test whether miR‐15a can directly bind to the 3′ untranslated region (UTR) of CD25 gene, we performed a dual luciferase assay (Figure 6G,H). Data showed that miR‐15a significantly reduced the luciferase activity of the wild‐type CD25‐3′UTR gene, rather than the mutant CD25‐3′UTR gene (Figure 6H), indicating that miR‐15a specifically bound to the 3′UTR of CD25 gene and suppressed its expression. Taken together, our findings indicate that NPC exosomal miR‐15a can induce γδT‐17 cells by downregulating CD25 expression.

## 3 DISCUSSION

In this study, we identified a significant presence of γδT‐17 cells in NPC tumor tissues and demonstrated that these γδT‐17 cells contributed to radioresistance in NPC, both in vitro and in vivo. Inhibiting IL‐17 secretion from γδT‐17 cells restored NPC radiosensitivity and enhanced radiation‐induced cell death. The upregulation of IL‐17‐promoting cytokines by NPC‐Exos in DCs, alongside the suppression of CD25/IL‐2 signaling in γδ‐T cells, facilitated the differentiation of γδT‐17 cells. Moreover, exosomal miR‐15a from NPC cells played a role in inhibiting CD25/IL‐2 signaling in γδ‐T cells. Our findings reveal a novel immunoregulatory function of NPC‐Exos, with potential therapeutic implications for overcoming radioresistance in NPC patients.

Although γδ‐T cells have demonstrated with promising antitumor activities, their functions can be diminished in the TME and even be educated into protumor phenotypes.^34^ For example, gastric cancer‐derived exosomal miR‐135b‐5p impairs the function of Vγ9Vδ2 T cells by targeting specificity protein 1.^35^ In addition, breast cancer‐derived exosomes transmit lncRNA SNHG16 to induce CD73^+^γδ1 Treg cells.^36^ Microenvironmental oxygen pressure also orchestrated pro‐tumoral γδ T cell via tumor‐derived exosomes by increasing the immunosuppressive functions of MDSCs.^37^ Previous studies have proved that γδT‐17 could promote tumor angiogenesis ^38^ or conspire with tumor‐associated neutrophils to promote metastasis.^39^ However, the role of γδT‐17 cells in NPC tumorigenesis remains unclear.

Although dual roles of IL‐17‐producing cells have been demonstrated in tumorigenesis and prognosis,^40^ more and more recent studies identified IL‐17‐producing cells as protumor factors through different pathways, including the promotion of tumor cell proliferation, migration, and angiogenesis.^39^, ^41^ IL‐17 could promote NPC cell proliferation and migration.^42^, ^43^ Although radiotherapy is the first‐line treatment for NPC and γδ‐T cells are enriched in nasopharyngeal tissues, it remains unknown about the effects of γδT‐17 cells on NPC radiotherapy. In our study, we found that IL‐17 secreted from NPC‐Exos‐induced γδT‐17 cells significantly upregulated the expression of anti‐apoptotic protein BCL‐2, which could promote radioresistance of cancer cells.^26^, ^27^ Blockage of γδT‐17 cells not only recovered the radiation‐induced NPC cell death in vitro but also improved the therapeutic efficacy of radiotherapy against NPC tumors in vivo. Since the pro‐metastatic effect of IL‐17 on NPC cells has been proved,^42^ γδT‐17 cells could also promote radioresistance by inducing NPC cell migration to escape from the irradiated area. In addition, IL‐17 can modulate DNA repair‐related gene expression, such as p53, to avoid radiation‐induced apoptosis and promote radioresistance of tumor cells.^44^ Therefore, our study provides a potential therapeutic approach by targeting γδT‐17 cells to improve NPC radiosensitivity.

Tumor exosomes are critical immunoregulators in the TME. They can regulate accessory immune cells and affect antitumor effector cells through the carried contents like protein and nuclear acid.^45^, ^46^ We found that NPC‐Exos induced robust secretion of IL‐17‐promoting cytokines (IL‐1β, IL‐6, and IL‐23) from antigen‐presenting cells, which are initiators for the induction of γδT‐17 cells. In addition, NPC‐Exos directly regulate the cytokine responsiveness of γδT‐17 cells to IL‐2 by downregulating CD25. It has been proved that CD25/IL‐2 signaling can polarize T‐cell differentiation into Treg phenotype by inducing the transcriptional factors of STAT5.^47^ In addition, CD25 signaling can activate STAT4 and polarize naïve T cells into Th1 cells. The repression of CD25/IL‐2 signaling downregulates STAT5 and STAT4 while upregulates STAT3 and further promotes T‐cell differentiation toward Th17 phenotype.^48^, ^49^, ^50^

Exosomes generally contain abundant miRNA and are the most important carriers of their circulating format.^51^ As critical mediators of intercellular communication in the TME, exosomes can regulate tumorigenesis, metastasis, and therapeutic responses by delivering miRNA.^52^ Multiple effects of miR‐15a have been reported in cancer biology previously. For example, miR‐15a downregulated the SLC29A7‐mediated Wnt/β‐catenin pathway to inhibit the tumorigenesis of prostate cancer.^53^ miR‐15 also promoted the malignant phenotypes of colorectal cancer by increasing PTEN/AKT and STAT3/TWIST1 signaling pathways.^54^ In addition, chemoresistance could be conferred to acute myeloid leukemia by miR‐15a by suppressing autophagy induced by daunorubicin.^55^ However, the direct immunoregulatory effects of miR‐15a on T‐cell differentiation have rarely been reported. In this study, for the first time, we found that tumor‐derived miR‐15a from NPC effectively promoted γδT‐17 cell induction. By targeting the 3′UTR of the CD25 gene, miR‐15 significantly downregulated CD25 expression and thus inhibited CD25 signaling to promote γδT‐17 cell induction. miR‐15a inhibitor successfully suppressed the induction of γδT‐17 cells by NPC‐Exos. Considering the promotion of NPC radioresistance by γδT‐17 cells, targeting tumor‐exosomal miR‐15a could be a potential therapeutic strategy to improve radiotherapy against NPC. In addition, some other types of cancer also express abundant miR‐15a, such as lung and prostate cancers,^56^ thus their exosomes have potentials to induce γδT‐17 cells as well. On the contrast, we found that exosomes from immortalized normal epithelial cells had minimal miR‐15a and did not promote γδT‐17 cells induction. Therefore, targeting exosomal miR‐15a‐γδT‐17 cells axis can be a promising approach to overcome radioresistance in cancer therapy.

In summary, we revealed a novel cell–cell communication pathway among NPC and γδ‐T cells. Abundant miR‐15a in NPC‐Exos can promote γδT‐17 cell induction and confer radioresistance to tumor cells. This novel route not only helps better understand the immunoregulatory mechanism of NPC but also provides implications for developing new approaches to overcome radioresistance in NPC patients.

## 4 METHOD

### 4.1 Cell culture

As mentioned in our earlier research,^57^ the NPC cell lines NPC43 and C666‐1 were kindly provided by Professor S.W. Tsao from The University of Hong Kong. C666‐1 cells were grown in RPMI medium supplemented with 10% fetal bovine serum (FBS), while NPC43 cells were maintained in RPMI with 10% FBS and 4 µM Y27632 (Enzo Life Sciences). NP69, an immortalized nasopharyngeal epithelial cell line, was cultured using keratinocyte serum‐free medium (ThermoFisher Scientific). HEK 293T cells were cultured in Dulbecco's modified Eagle medium (DMEM) supplemented with 10% FBS.

Ionizing radiation was administered using a Gammacell 3000 irradiator (Nordion International). All experimental protocols were reviewed and approved by the Institutional Review Board of The University of Hong Kong/Hospital Authority Hong Kong West Cluster, as well as the Committee on the Use of Live Animals in Teaching and Research at The University of Hong Kong.

### 4.2 Exosomes isolation

Exosomes were purified using differential ultracentrifugation at 4°C, following established protocols.^24^, ^57^, ^58^ Initially, the conditioned medium was centrifuged at 300 × *g* for 10 min, then at 2000 × *g* for another 10 min, and finally at 10,000 × *g* for 30 min. The supernatant was filtered using a 0.22‐µm syringe filter and ultracentrifuged at 100,000 × *g* for 70 min with an SW32Ti rotor (Beckman). The pellet was resuspended in PBS and subjected to a second round of ultracentrifugation at 100,000 × *g* for another 70 min. The final exosomal pellet was resuspended in PBS. For cellular studies, exosomes were applied at a concentration of 20 µg/mL, with protein levels tested using a BCA Protein Assay Kit (Pierce).

### 4.3 Immunofluorescent analysis

NPC tumor tissues were fixed in 10% formalin and embedded in paraffin for sectioning. The infiltration of human γδT‐17 cells was assessed through immunofluorescence using anti‐human IL‐17 (R&D Systems) and TCR‐γδ (Biolegend) antibodies. Images were acquired using an LSM 710 Confocal Microscope (Zeiss, Germany).

### 4.4 Induction of γδT‐17 cells

Human peripheral blood mononuclear cells (PBMCs) were isolated from healthy donors and subjected to γδ‐T cells purification via TCRγ/δ+ T Cell Isolation Kit (Miltenyi). Then, purified γδ‐T cells were treated with NP69‐Exos or NPC‐Exos and cultured in the presence of DCs or 50% supernatant from DCs pretreated with or without NPC‐Exos. Seven days later, the expression of IL‐17 in γδ‐T cells was determined by flow cytometry. In some experiments, purified γδ‐T cells were treated with NPC‐Exos and cultured with 50% supernatant from NPC‐Exos‐pretreated DCs, in the presence of IL‐2, neutralizing anti‐IL‐1beta, anti‐IL‐6, anti‐IL‐23, anti‐CD25 antibody, or isotype controls (eBioscience). To collect the culture supernatant of γδT‐17 cells for downstream experiments, the cells were restimulated with anti‐CD3/CD28 beads (Miltenyi) overnight.

### 4.5 Cell apoptosis assay

NPC cells were exposed to 3 Gy of radiation and cultured with varying concentrations (0, 10, 50, or 100 ng/mL) of IL‐17 or 50% supernatant from γδT‐17 cells, in the presence of neutralizing anti‐IL‐17 antibody (R&D Systems) or isotype control. After 48 h, cell apoptosis was measured using the Annexin V Apoptosis Detection Kit (Biolegend). In some experiments, BCL‐2 expression was determined in permeabilized NPC cells after 24 h.

### 4.6 Establishment and treatment of NPC tumors in Rag2^−/−^γc^−/−^ mice

C57BL/10SgAiRag2^−/−γc−/−^ mice were housed in the Laboratory Animal Unit at The University of Hong Kong. NPC tumor models were established following previously described protocols.^57^, ^59^, ^60^ Briefly, C666‐1 cells were combined with an equal volume of Matrigel (Corning) and injected subcutaneously into 6‐ to 8‐week‐old Rag2^−/−γc−/−^ mice. Mice with palpable tumors were randomly divided into groups. They were irradiated with either 0 or 4 Gy, and supernatants from γδT‐17 cells (50 µg/mouse) were administered every 3 days for five doses, with or without 15 µg/mouse neutralizing anti‐IL‐17 antibody or isotype control (R&D Systems). Equivalent volumes of complete culture medium were used as a control for the γδT‐17 cell supernatant. After 3 weeks, the tumors were excised and their size measured. Tumor volume was calculated as length × [width]^2^ × 0.52. In accordance with regulations at The University of Hong Kong, mice were sacrificed once their tumor diameters reached 17 mm. At study endpoints, tumor tissues were excised, photographed, and processed for immunohistochemical analysis.

### 4.7 Cellular uptake of NPC‐Exos

NPC‐Exos were labeled with carboxyfluorescein succinimidyl ester (CFSE; Sigma‐Aldrich) to track cellular uptake. Excess dye was removed by re‐centrifugation at 100,000 × *g* for 70 min, performed twice. As a control, pellets were isolated from non‐conditioned FBS‐exosome‐free medium and similarly labeled with CFSE. Cells were incubated with CFSE‐labeled NPC‐Exos or control for 24 h, after which cellular uptake was assessed via flow cytometry or immunofluorescence analysis.

### 4.8 Secretion of IL‐17‐promoting cytokines from DCs

Monocyte‐derived DCs were cultured as described in previous studies.^58^ The DCs were treated with NPC‐Exos and cultured for 48 h. The culture supernatant was then collected and analyzed for IL‐17‐promoting cytokines. For subsequent experiments, DCs from four donors were treated with or without NPC‐Exos, and their culture supernatants were collected and mixed after 48 h for further use.

### 4.9 Flowcytomix assay

Cytokine concentrations in the culture supernatants were detected and analyzed using the LEGENDplex Human Inflammation or Th panel kit, in accordance with the manufacturer's instructions (Biolegend).^61^

### 4.10 Expression of cytokine receptors

Human PBMCs were isolated from healthy donors and subjected to γδ‐T cells purification via TCRγ/δ+ T Cell Isolation Kit (Miltenyi). Then, purified γδ‐T cells were treated with NP69‐Exos or NPC‐Exos and cultured in the presence of anti‐CD3/CD28 beads (Miltenyi). Forty‐eight hours later, the expressions of CD25, IL‐1R1, IL‐6R, and IL‐23R were detected by flow cytometry.^62^

### 4.11 miRNA sequencing and qPCR

Small RNAs were extracted from NP69‐Exos and C666‐1‐Exos using the exoRNeasy kit (Qiagen). CDNA libraries were prepared with the QIAseq miRNA kit (Qiagen) and sequenced on the Illumina HiSeq TM 3000/4000 platform at Epibiotek. FastQC ensured read quality, and DESeq2 identified differentially expression (log2 fold change > 1, false discovery rate < 0.05). Heatmaps and volcano plots were generated using pheatmap and ggplot2.

The isolated RNA was reverse transcribed into cDNA to assess miRNA expression using the miRCURY LNA RT Kit (Qiagen). Real‐time PCR was carried out with the miRCURY LNA SYBR Green PCR kit, and the results were analyzed on an ABI7900 system (Applied Biosystems). U6 was used as the internal control, and relative expression levels were calculated using the 2^−ΔΔCT^ method. To evaluate the transfer of exosomal miR‐15a, γδ‐T cells were treated with NP69‐Exos or NPC‐Exos in the presence of DMSO or actinomycin D, and the miR‐15a expression levels were measured 24 h later.

### 4.12 Luciferase assay

Interaction between miR‐15a and CD25 was determined using a dual luciferase assay. The pmirGlo Dual‐Luciferase miRNA Target Expression Vector (Promega) was utilized to construct plasmids carrying either the wild‐type (CD25‐WT‐3′ UTR) or mutated (CD25‐Mut‐3′ UTR) versions of the CD25 3′ UTR. These plasmids were co‐transfected into HEK293T cells along with either a miR‐15a mimic or a negative control. Dual‐Glo Luciferase Assay kit (Promega) was used to determine luciferase activity 24 h post‐transfection.

### 4.13 Statistical analysis

Quantitative results are presented as mean ± standard error of the mean (SEM). The Mann–Whitney *U* test was applied for comparisons between two groups, while one‐way analysis of variance with Bonferroni correction was used for multiple group comparisons. All tests were two‐tailed, and a *p* value below 0.05 was considered statistically significant.

## AUTHOR CONTRIBUTIONS

W.T. and X.W. conceptualized and designed the study, interpreted the findings, and contributed to writing and editing the manuscript. X.W. and Z.X. were responsible for designing and conducting the experiments, analyzing the data, and drafting the manuscript, with support from Z.X., Y.Z., C.R.T., C.H., Y.C., W.Z., M.W., and Y.L. All authors have read and approved the final manuscript.

## CONFLICT OF INTEREST STATEMENT

The authors declare no conflicts of interests.

## ETHICS STATEMENT

All research protocols, animal procedures, and experiments involving human participants in this study received approval from the Institutional Review Board of The University of Hong Kong/Hospital Authority Hong Kong West Cluster (reference number UW10‐481) and the Committee on the Use of Live Animals in Teaching and Research at The University of Hoang Kong (No. 3989‐16). Written informed consent was obtained from all participants.

## Supporting information



Supporting Information

## Data Availability

The raw scRNA‐seq data in this study have been deposited to Genome Sequence Archive (GSA) in BIG Data Center, Beijing Institute of Genomics (BIG) under accession number CRA 013543.
