# Supplementary material for: CD25 downregulation by tumor exosomal microRNA‐15a promotes interleukin‐17‐producing γδ‐T‐cells‐mediated radioresistance in nasopharyngeal carcinoma
Source: MedComm (2020). 2025 Feb 2;6(2):e70078. doi: 10.1002/mco2.70078 (PMC11788015; doi:10.1002/mco2.70078)
Supplement: Supplementary file 1 — Supporting Information [file MCO2-6-e70078-s001.docx]

**CD25 downregulation by tumor exosomal microRNA-15a promotes interleukin-17-producing γδ-T cells -mediated radioresistance in nasopharyngeal carcinoma.**

Xiwei Wang^1,2^, Zheng Xiang^1,3^, Yanmei Zhang^1,2^, Chloe Ran Tu^4^, Chunyu Huang^5^, Yuet Chung^1^, Wenyue Zhang^1^, Manni Wang^1^, Yinping Liu^1^ and Wenwei Tu^1,2^*

^1^Department of Paediatrics & Adolescent Medicine, Li Ka Shing Faculty of Medicine, University of Hong Kong, Hong Kong SAR, China

^2^CAS Key Laboratory of Quantitative Engineering Biology, Shenzhen Institute of Synthetic Biology, Shenzhen Institute of Advanced Technology, Chinese Academy of Sciences, Shenzhen 518055, China.

^3^Department of Microbiology and Immunology, Health Science Center (School of Medicine), Jinan University, China.

^4^Department of data sciences, Dana-Farber Cancer Institute, Harvard University, Boston, Massachusetts, USA.

^5^Shenzhen Key Laboratory for Reproductive Immunology of Peri-implantation, Shenzhen Zhongshan Institute for Reproduction and Genetics, Shenzhen Zhongshan Obstetrics & Gynecology Hospital (formerly Shenzhen Zhongshan Urology Hospital), Shenzhen, Guangdong, China.

*Corresponding author.

Wenwei Tu, Department of Pediatrics and Adolescent Medicine, the University of Hong Kong, Room L7-56, 7/F Laboratory Block, Faculty of Medicine Building, 21 Sassoon Road, Hong Kong; Phone: (852) 3917 9354; Fax: (852) 2819 8142; E-mail: wwtu@hku.hk



**Supplementary figures & legends**


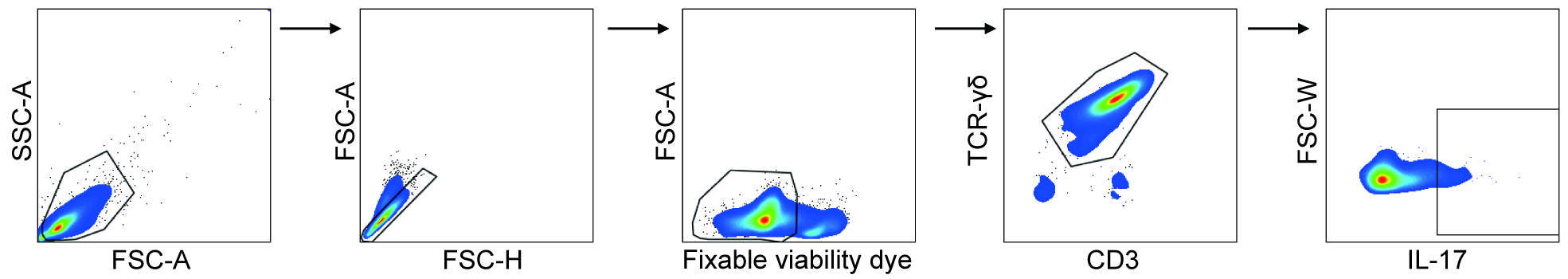


Fig. s1 Gating strategy of γδT-17 cells

γδ-T cells were purified from PBMCs using magnetic beads and treated with NPC-Exos. After 7 days, cells were collected and subjected to detection using flow cytometry. The data shown are representatives from four biological replicates.


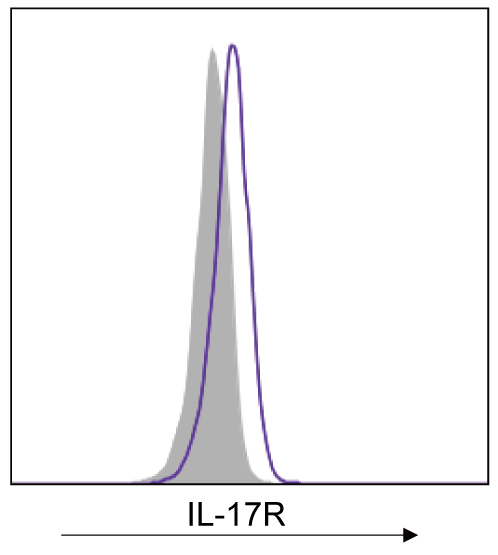


Fig. s2 Expression of IL-17 receptor on immortalized normal epithelial cells.

Expression of IL-17R on immortalized normal epithelial cells (NP69) was detected by flow cytometry. The data shown is a representative from three independent experiments.


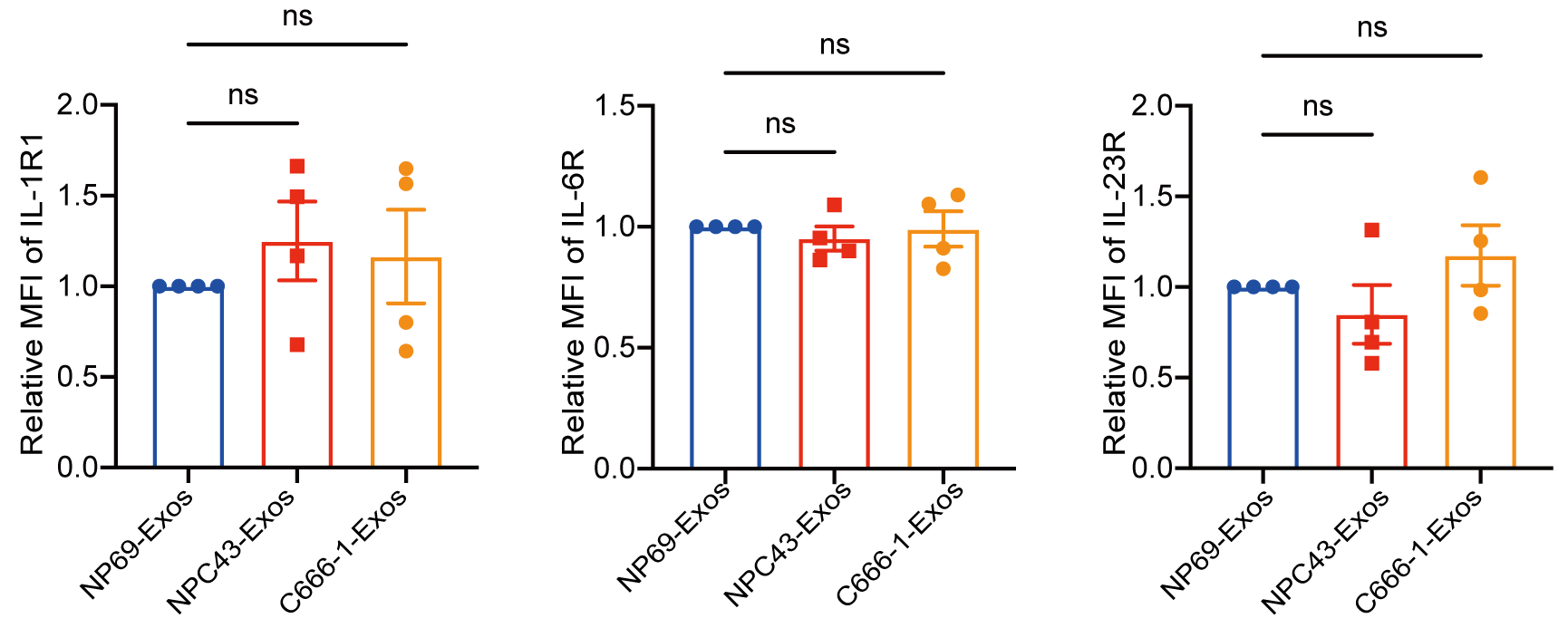


Fig.s3 Effects of NPC-Exos on the receptors of IL-17-driving cytokines.

Purified γδ-T cells were treated with NP69-Exos or NPC-Exos and cultured in the presence of anti-CD3/CD28 beads. 48 hours later, the receptors of IL-1β, IL-6 or IL-23 on γδ-T cells were detected. Quantitative data are shown as mean ± SEM of four biological replicates. ns, not significant.


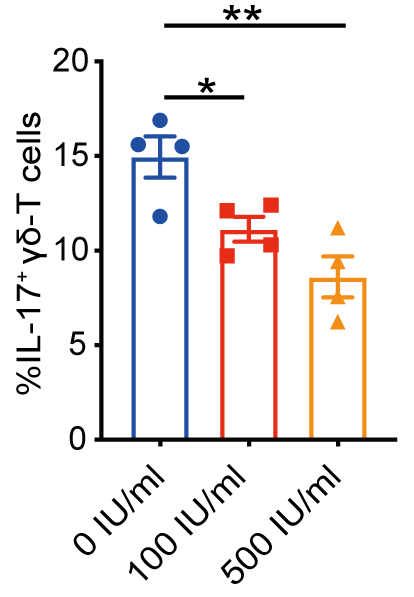


Fig. s4 IL-2 inhibits the induction of γδT17 cells by NPC-Exos.

γδ-T cells were treated with C666-1-Exos and cultured with supernatant from C666-1-Exos-pretreated DCs containing 0, 100 or 500 IU/ml IL-2. Seven days later, the percentages of γδT-17 cells were determined. Quantitative data are shown as mean ± SEM of four biological replicates. *p < 0.05, **p < 0.01.
